# Traumatic spinal fracture treated by vertebroplasty: a case report

**DOI:** 10.1186/1752-1947-6-390

**Published:** 2012-11-21

**Authors:** Gabriel Claudiu Tender, Daniel Serban

**Affiliations:** 1 Department of Neurosurgery, Louisiana State University, 2020 Gravier Street, Suite 744, New Orleans, LA, USA; 2 Department of Neurosurgery, ‘Bagdasar-Arseni’ Hospital, Sos. Berceni, Nr. 12, Sector 4, Bucharest, 041915, Romania

**Keywords:** Lumbar burst fracture, Spinal mobility, Vertebroplasty

## Abstract

**Introduction:**

Surgical treatment for lumbar burst fractures is complex and typically involves either a retroperitoneal corpectomy and/or a posterior pedicle screw fixation. We describe the case of a patient with a lumbar burst fracture who was cured via a less invasive approach that has not been previously reported as standalone treatment.

**Case presentation:**

This 25-year-old Caucasian man presented with excruciating axial low back pain exacerbated by any attempt to elevate the head of the bed after a motor vehicle accident. Computed tomography demonstrated a burst L4 fracture without spinal canal compromise. The patient underwent a bilateral vertebroplasty with an injectable polymer that mimics cortical bone. Postoperatively, the patient was progressively mobilized in a thoracolumbar spinal orthosis brace without any recurrence of pain. Postoperative computed tomography showed no loss of height in the L4 vertebral body. At one-year postoperatively, the patient was symptom free and the computed tomography scan showed good fracture healing.

**Conclusion:**

Retroperitoneal corpectomy and/or posterior multi-segment fixation for lumbar burst fractures without neural compression in young patients are associated with loss of mobility and potential future adjacent level disease. Our limited vertebroplasty intervention with close postoperative clinical monitoring has not been previously described as standalone treatment, and it offers the advantages of less operative morbidity and maintenance of lumbar mobility in selected patients.

## Introduction

There is considerable controversy regarding the treatment of lumbar burst non-osteoporotic fractures without neurological deficit [1]. Surgical treatment typically involves either a retroperitoneal corpectomy and/or a posterior pedicle screw fixation. The surgical indication and choice of technique depend on multiple factors, including the severity of symptoms, the degree of vertebral body height loss and canal compromise, and the integrity of the posterior spinal elements [2]. We describe the case of a patient with lumbar burst fracture who was cured via a less invasive approach that has not been previously reported as standalone treatment.

## Case presentation

A 25-year-old Caucasian man presented to the emergency room, after a motor vehicle accident, with excruciating axial low back pain (score of 10 out of 10 on visual analog scale) that was improved by lying in a supine position and exacerbated by any attempt to elevate the head of the bed. On physical examination, he exhibited local tenderness to palpation and percussion, but no neurological deficit nor any signs of radiculopathy. An attempt was made to mobilize the patient in a thoracolumbar spinal orthosis device (TLSO), but the pain precluded any elevation of the upper body above 30 degrees. Laboratory data were unremarkable.

**Figure 1:**
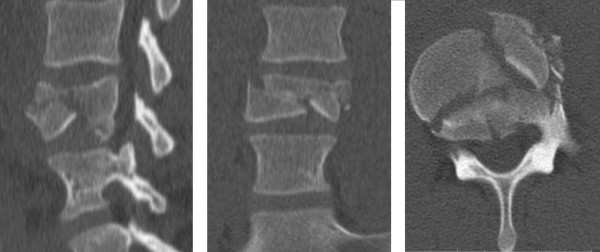
**Preoperative computed tomography imaging.** There is a lumbar L4 burst fracture with minimal loss of height and canal compromise.

Plain radiographs and noncontrast lumbar computed tomography (CT) demonstrated a burst L4 fracture, Magerl type A3.3, with minor loss of vertebral body height and without spinal canal or neuroforamina compromise (Figure 1). Noncontrast lumbar magnetic resonance imaging (MRI) demonstrated integrity of the posterior spinal elements.

The patient could not benefit from a conservative management trial, including mobilization and physical therapy in a TLSO brace, because any attempts to elevate the head of the bed resulted in excruciating pain at the site of the fracture.

The severity of symptoms and the presence of the L4 fracture prompted the decision to proceed with surgical treatment. The option of total bed rest without any mobilization for several months, until the fracture would start to heal, was considered unacceptable by both the patient and the physicians. The following surgical options were presented to the patient:

1. Retroperitoneal approach, L4 corpectomy and L3–L5 fixation with anterior titanium cage supplemented with either lateral or posterior instrumentation.

2. Posterior midline approach, L4 vertebroplasty, L3–L5 pedicle screw fixation, and posterior and postero-lateral bone grafting with the expectation to achieve a fusion.

3. Posterior midline approach, L4 vertebroplasty, and temporary L3–L5 pedicle screw fixation, with the expectation to remove the instrumentation after the expected L4 fracture healing. The presence of a posterior cortical breach was not considered a contraindication for the vertebroplasty.

4. L4 vertebroplasty as a first step, followed by temporary L3–L5 pedicle screw fixation as a second operation, if the pain remained incapacitating and/or if any neurological symptoms occurred with mobilization.

**Figure 2:**
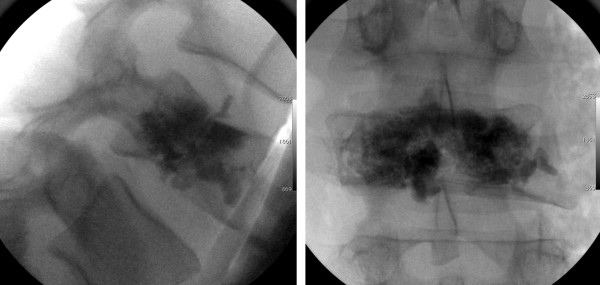
**Intraoperative radiographic imaging.** Antero-posterior and lateral roentgenograms demonstrate the polymer injected into the vertebral body (i.e., the vertebroplasty).

The patient decided to proceed with the standalone L4 vertebroplasty (option 4) in order to minimize the surgical morbidity and preserve lumbar mobility. The goal of surgery was to provide sufficient anterior spinal structural support, as measured by pain and/or neurological symptoms with mobilization, until the expected L4 fracture healing. The operation was performed the day after the accident. The patient underwent a bilateral vertebroplasty using CORTOSS™ (Orthovita, Malvern, PA, USA), an injectable, non-resorbable polymer that mimics cortical bone (Figure 2). The vertebroplasty trocar was repositioned one time through the left L4 pedicle in order to augment the multiple lines of fracture on that side. The polymer choice was dictated by the similarity to the cortical bone and the better assimilation by the vertebral body, when compared to polymethylmethacrylate (PMMA).

**Figure 3:**
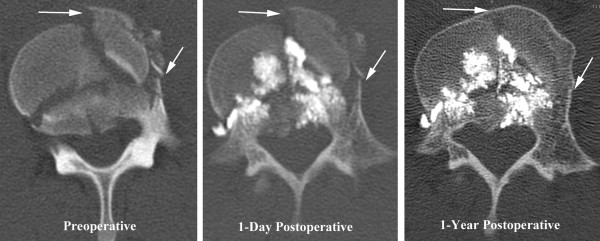
**Preoperative, one-day postoperative, and one-year postoperative axial computed tomography imaging.** These axial images demonstrate bone growth at the fracture lines (arrows).

The patient reported minimal residual pain immediately after the surgery. He was progressively mobilized in a TLSO brace, initially to a semi-sitting position, and then to a sitting and standing position, without any recurrence of pain. Multiple CT scans performed in the first postoperative week showed no loss of height in the L4 vertebral body. The patient was able to ambulate independently on the fourth postoperative day and was discharged on the fifth postoperative day with the recommendations to use the TLSO brace whenever in an upright position, for a total of three months, and to return to the hospital for any recurrence of pain or new neurological symptoms. At one-year postoperatively, the patient was symptom free and the CT scan showed good fracture healing (Figure 3) with no further height loss of the vertebral body.

## Discussion

Early studies emphasized the importance of anterior support for unstable fractures [3]. Although an anterior retroperitoneal approach is best employed when spinal canal decompression is desired, it requires an access surgeon and may be associated with significant morbidity [4].

In order to minimize morbidity, posterior approaches have been described to achieve both the lumbar corpectomy and the posterior fixation [5]. Other studies focused on posterior instrumentation alone. Although short-segment posterior fixation appears insufficient in the absence of anterior support [6], extension of the posterior fusion to two segments above and one below the fracture may prove successful, at the cost of decreased mobility [7]. In an attempt to reduce the number of fused segments, other authors employed placement of a screw in the fractured vertebral body [8] or the addition of vertebroplasty augmentation.

A prospective study comparing spinal fusion versus temporary fixation for the treatment of burst fractures favored the former [9]. Other authors combined vertebroplasty with a temporary short-segment fixation, with removal of instrumentation at one year, with good results [10].

Several articles have described the use of standalone vertebroplasty for the treatment of lumbar fractures [11-13]. Our patient’s treatment was different from all the patients described in these articles in several ways. First, the authors in all the cited articles used cement (calcium phosphate cement or PMMA), whereas we used a synthetic cortical bone substitute (CORTOSS™) that has viscous properties allowing for slow and safe injection, which is much better integrated by the host bone. These handling and biological features of CORTOSS™ permitted not only the safe injection in a fractured vertebral body with a posterior cortical breach, but also a seamless distribution and incorporation into the fracture lines. Second, the presentation and indications for treatment in our patient were radically different from the ones in the cited articles. Our patient had a comminuted L4 burst fracture resulting in excruciating pain immediately after the accident on even minor attempts to elevate the head of the bed. Therefore, he could not be mobilized in any way, and ‘conservative treatment’ would have implied keeping him flat in bed for at least several months until the fracture had started to heal. This option was considered unacceptable by both the patient and the physicians. The patients in the cited studies underwent successful mobilization to at least sitting at the side of the bed, and the vertebroplasty was performed at a relatively long time after the fracture, when the bone was probably at least partially healed.

Finally, there are several authors who believe there is no benefit from surgical versus conservative treatment for these patients [14-16].

The approach we used in this case – a standalone vertebroplasty for the treatment of non-osteoporotic lumbar burst fractures, Magerl type A3.3 – represents an alternative strategy that complements those already presented. This approach offers the advantages of minimal operative morbidity and preservation of lumbar mobility, and may be supplemented, if necessary, by subsequent short-segment pedicle screw fixation and/or fusion.

We prefer to use vertebroplasty instead of kyphoplasty in patients with fractures that do not result in significant focal kyphosis, because balloon inflation during the kyphoplasty entails an increased risk of the displacement of fracture fragments into the spinal canal. Typical cementoplasty risks, such as extravasation into the spinal canal or venous system, or thermal effects during the polymerization of PMMA, are decreased or absent when synthetic cortical bone material is used.

## Conclusion

Current surgical options for lumbar burst fractures without neural compression are associated with loss of mobility and potential future adjacent level disease, which may be unacceptable in young patients. We describe a limited vertebroplasty treatment with close postoperative clinical monitoring that has not been previously described as standalone treatment for this type of fracture, and it offers the advantages of less operative morbidity and maintenance of lumbar mobility in selected patients.

## Consent

Written informed consent was obtained from the patient for publication of this case report and accompanying images. A copy of the written consent is available for review by the Editor-in-Chief of this journal.

## Competing interests

The authors declare that they have no competing interests.

## Authors’ contributions

GT and DS analyzed the patient’s data and were major contributors in writing the manuscript. GT performed the surgical intervention. Both authors read and approved the final manuscript.
